# Special issue: frontiers in recent advances on cancer diagnosis and treatment

**DOI:** 10.48101/ujms.v129.11919

**Published:** 2024-12-31

**Authors:** Bengt Westermark, Carl-Henrik Heldin

**Affiliations:** a Department of Immunology, Genetics and Pathology, Uppsala University, Uppsala, Sweden; b Department of Medical Biochemistry and Microbiology, Uppsala University, Uppsala, Sweden

‘Of all the processes that the student of pathology is privileged to study, none is as intriguing, fascinating, and perplexing as neoplasia.’

This statement, a quote from William Boyd’s *Textbook of Pathology* (1961), remains as thought-provoking today as when it was first written. Over the decades since Boyd penned these words, our understanding of the molecular mechanisms underlying tumorigenesis and the development of novel therapeutic approaches have advanced at a revolutionary pace. This special issue of *UJMS* on cancer exemplifies the remarkable progress in cancer research, reaffirming that Boyd’s perspective on the enigmatic nature of cancer endures.

The principles underlying malignant transformation and oncogenesis have been extensively summarized and conceptualized by Douglas Hanahan and Robert Weinberg in their landmark publications on the hallmarks of cancer (1). The articles featured in this issue of UJMS provide focused insights into some of the hallmarks presented in the most recent version of these seminal works (2).

Mutations in the *TP53* tumor suppressor gene are among the most common genetic alterations in human cancers. Efforts to target the TP53 pathway are advancing, with one of the most promising approaches presented by Strandgren and Wiman (3). Their study highlights cases where mutations produce a truncated, non-functional protein due to a premature stop codon. They explore strategies to induce readthrough of the mutated codon, enabling the generation of a full-length protein with restored functionality. Although this approach addresses only about 10% of TP53 mutations, it represents a significant step forward in the effort to develop targeted cancer therapies.

Andersson (4) conducted a novel study on the relationship between hair graying and the development of melanoma in horses. The research identified the Grey allele within an intron sequence of the Syntaxin 17 gene. This Grey mutation was attributed to a sequence multiplication, resulting in two or three additional copies of the sequence. While the specific function of these duplicated sequences remains unclear, they appear to be associated with a weak enhancer effect and the upregulation of candidate cancer-causing genes, including the orphan nuclear receptor NR4A3. Further functional studies on NR4A3 could provide deeper insights into melanoma development, both in horses and humans.

The review by Mendes-Rodrigues-Junior and Moustakas (5) highlights the role of TGF-β-induced long non-coding RNAs (lncRNAs) as key regulators of epigenetic, transcriptional, and post-transcriptional processes across various human malignancies. TGF-β has a dual role in cancer; initially it is a tumor suppressor as it inhibits the growth of most cell types and induces apoptosis, but in advanced cancer TGF-β instead promotes tumorigenesis, e.g. by stimulating epithelial-mesenchymal transition that promotes invasion and metastasis. Several lncRNAs modulate the tumor suppressing or tumor-promoting effects of TGF-β, underscoring the intricate complexity of regulation of TGF-β signaling in cancer.

Four reviews explore the complex interactions between cancer cells and the tumor stroma. Pietras and Sjölund (6) provide compelling data on how tumor cell plasticity is influenced by signals from cancer-associated fibroblasts and infiltrating macrophages. Additionally, single-cell RNA sequencing reveals unexpected heterogeneity and spatial organization within tumor-infiltrating cells. As the authors emphasize, unraveling the dynamics of the cancer ecosystem could offer valuable insights into the development of more effective treatments.

Milosevic and Östman (7) present a fascinating perspective on tumor-associated fibroblasts. Specific subsets of these cancer-associated fibroblasts appear to have unique abilities to modulate the functions of infiltrating T cells and antigen-presenting cells, thereby contributing to the immunosuppressive tumor microenvironment. This insight opens up new opportunities for identifying prognostic and potentially predictive biomarkers, as well as novel therapeutic targets.

Badillo et al. (8) review the subtypes of tumor-infiltrating dendritic cells (DCs) and their capacity to present tumor antigens to T cells. They explore how the tumor immune microenvironment influences the plasticity and function of these DCs. These insights may pave the way for the development of innovative DC-based antitumor therapies.

Sjöberg (9) focuses on the role of the endothelium in the micro-environment of clear cell renal cell carcinoma (ccRCC). Multi-omics methods, including single cell analyses, have revealed distinct endothelial cell types in ccRCC of potential importance for disease progression, patient prognosis and therapy prediction.

In his review, Glimelius (10) highlights a critical clinical issue regarding advancements in the treatment of rectal cancer. While significant progress has been made in improving locoregional tumor control, there remains a pressing need to enhance long-term survival outcomes. The author emphasizes the importance of developing more accurate risk prediction methods and optimizing systemic treatments to reduce systemic recurrences and extend patient survival.

Falkman et al. (11) present a recent report on the treatment of neuroendocrine tumors, utilizing mutational analysis. Among 12 patients analyzed through DNA sequencing, two were identified with targetable *BRAF* mutations. Treatment with a combination of BRAF and MEK inhibitors resulted in tumor size reduction. Unfortunately, the beneficial effects were transient. The authors suggest that DNA sequencing should be a routine in the clinical management of neuroendocrine tumors.

While the molecular genetics of glioblastoma has been extensively studied, much less attention has been given to meningioma, the most common brain tumor in adults. Szulzewsky et al. (12) publish an important report highlighting recent advances in the understanding of meningioma biology, including an analysis of its mutational landscape. Notably, the authors also discuss the development of engineered mouse models for studying meningioma.

Takahashi-Yamashiro and Miyazono (13) explore the application of tissue clearing methods as a powerful tool for analyzing experimental mouse models of cancer. Although these techniques have been predominantly used in neurobiology, the authors effectively demonstrate their potential for use in cancer research. In a metastatic cancer model, they showcase a resolution down to the single-cell level. Furthermore, spatial transcriptomics and protein expression analyses of cleared, tumor-infiltrated tissues offer promising new insights into the biology of tumor growth and metastasis.
